# Dimensional changes in buccal cortical bone and lesion volume in teeth with persistent chronic periapical disease subjected to periapical surgery: a cone beam computed tomography study at one year of follow-up

**DOI:** 10.4317/medoral.27006

**Published:** 2025-02-15

**Authors:** Araceli Boronat-López, Juan Carlos Bernabeu-Mira, Miguel Peñarrocha-Diago, María Peñarrocha-Diago, David Peñarrocha-Oltra

**Affiliations:** 1 Master in Oral Surgery and Implantology, Department of Stomatology, Faculty of Medicine and Dentistry, University of Valencia, Spain; 2 Full Professor, Department of Stomatology, Faculty of Medicine and Dentistry, University of Valencia, Spain; 3 Chairman, Department of Stomatology, Faculty of Medicine and Dentistry, University of Valencia, Spain

## Abstract

**Background:**

This study aimed to evaluate changes in buccal cortical bone and lesion volume in teeth with persistent periapical disease one year after periapical surgery using cone-beam computed tomography (CBCT).

**Material and Methods:**

A prospective study was conducted involving patients with persistent periapical disease undergoing periapical surgery, with one year of follow-up. Data collected included patient age, gender, teeth involved, and the number of roots/lesions. CBCT measurements were taken preoperatively and one year post-surgery, including the distance from the cementoenamel junction to the buccal bone crest (CEJ-BBC), marginal bone loss, buccal cortical height, presence of fenestration, apical depth, cortical bone width at 1, 3, and 5 mm from the buccal bone crest, and lesion volume in mm³. Success was assessed using the “Modified Penn 3D criteria.”

**Results:**

The study included 92 patients with 111 roots exhibiting persistent chronic periapical lesions. Statistically significant changes were observed in all buccal cortical bone parameters one year after surgery. The CEJ-BBC distance increased, indicating a marginal bone loss of 0.23 mm. Notably, the height from the buccal cortical bone crest to the lesion, apical depth, buccal bone thickness, the number of fenestrations, and lesion volume decreased (91.1%). Buccal cortical bone thickness was a predictor of volume reduction, showing a significant relationship at T1 between greater thickness and smaller volume variation. Patient age and gender did not significantly influence these changes. Fenestrations and larger lesion volumes correlated with reduced healing probabilities. The overall success rate was 88%, with tooth position and root involvement impacting healing outcomes.

**Conclusions:**

One year post-surgery, buccal cortical bone showed no clinically relevant changes, while lesion volume decreased by 91.1%, more significantly in anterior teeth. Greater buccal cortical bone width was associated with smaller volume reduction. A larger lesion volume and presence of fenestrations adversely affected healing rates.

** Key words:**Buccal cortical bone, bone width, CBCT, prospective study, periapical surgery.

## Introduction

The buccal cortical bone of the alveolar process undergoes continuous remodeling due to its sensitivity to external stimuli (1). The dimensions of the alveolar process depend on the location, size, and inclination of erupted tooth roots, with the buccal bone wall being thinner than the lingual/palatine wall (2,3). Periapical surgery for persistent periapical disease may affect the buccal cortical bone over time, influencing both the prognosis of the surgery and the lesion volume. The reference model for measuring buccal cortical bone is typically used in cadavers or post-extraction, which is not feasible when preserving the tooth. In this context, cone-beam computed tomography (CBCT) is the most widely used technique (4). Behnia *et al*. (5) noted CBCT’s limitations in precision when the buccal cortical bone measures less than 1 mm, often overestimating the thickness. However, Li *et al*. (6) found no significant differences between clinical measurements and CBCT for alveolar bone height and thickness.

While some studies have measured buccal cortical bone dimensions in healthy teeth (7-11), few have focused on teeth with apical disease (12,13). Understanding potential differences in cortical bone between healthy teeth and those with apical disease is important. Measurements can be made from various anatomical reference points, such as the buccal bone crest (7-10) or cementoenamel junction (CEJ) (11,14). The distance from the amelocemental line to the vestibular bone crest (CEJ-BBC) varies from 1.6 to 3.40 mm based on tooth type (7,12-15). Additionally, vestibular cortical bone width varies by measurement points; Wang *et al*. (15) found this measurement to be less than 1 mm in 80% of anterior teeth and 40% of premolars, consistent with findings by Zekry *et al*. (16) and Vera *et al*. (17).

The prognosis of periapical surgery varies among studies based on patient selection, treatment success scoring, and follow-up duration. Rubinstein and Kim (18) observed that 91.5% of teeth healed after one year remained healthy in long-term follow-up (5-7 years). Some authors suggest that larger preoperative lesions correlate with lower success rates (19), while others argue that volume does not influence success (6,20). Given the limited publications on buccal cortical bone changes in teeth with persistent periapical disease post-surgery, it is important to determine whether the buccal cortical bone serves as a predictor of healing and if periapical surgery affects its structure.

The present study was thus carried out to measure buccal cortical bone and lesion volume using CBCT in teeth with persistent periapical disease before and one year after periapical surgery. An evaluation was made of the changes produced and of the possible influence of patient age and gender, and the position and location of the tooth upon these measures. The null hypothesis for these objectives could be stated as follows: there is no significant difference in buccal cortical bone measurements and lesion volume, as assessed by CBCT, in teeth with persistent periapical disease before and one year after periapical surgery. Additionally, there is no significant impact of patient age, gender, tooth position, and location on these measurements.

Material and methods

- Study design

This prospective case series study was carried out in the Department of Oral Surgery (Faculty of Medicine and Dentistry, University of Valencia, Valencia, Spain) between January 2020 and December 2022.

The study design was approved by the Ethics Committee of the University of Valencia (Ref. number: 1224932) and abided with the principles of the Declaration of Helsinki (21). All patients were free to withdraw from the study at any time. The procedures were undertaken with the understanding and written consent of each subject. The present manuscript is presented following the STROBE statement (22).

- Patient selection

Patients presenting a single tooth with a persistent chronic periapical lesion subjected to periapical surgery were included in the study. The patients were informed about the surgical procedure, and a full medical history was compiled, with the recording of clinical and tomographic parameters (before and 12 months after surgery). The exclusion criteria were minor patients (< 18 years), teeth with endoperiodontal lesions, cases of apical cracks, teeth in which bone substitutes or grafts were used to fill the bone cavity, cases where the palatal root was affected and/or the surgical access was better through the palate and teeth without postoperative tomographic control or with poor quality images.

- Surgical technique

Antibiotic prophylaxis was prescribed in all cases in the form of 2 g of amoxicillin or 600 mg of clindamycin (in patients with allergy to penicillin) one hour before surgery. All surgeries were performed by the same professional (MPD or DPO) under local infiltration anesthesia with 4% articaine and 1:100,000 epinephrine (Inibsa®, Lliça del Vall, Barcelona, Spain). Following the submarginal incision, a full thickness mucoperiosteal flap was raised and ostectomy was performed to access the root apexes and apical lesion. The affected root was sectioned approximately 3 mm from the apex using a minimum or zero bevel, with curettage of the diseased tissue. Cavity preparation at the extremity of the root was carried out with ultrasound tips (Piezomed®, W&H Dentalwerk, Bürmoos GmbH, Bürmoos, Salzburg, Austria) to a depth of about 3 mm, followed by filling with mineral trioxide aggregate (MTA) (ProRoot®; Dentsply Tulsa Dental, Tulsa, OK, USA). All surgeries were performed with an endoscope (Karl Storz-Endoskope®, Tuttlingen, Germany) for inspecting root sectioning, root preparation and retrograde filling. After cleaning the bone cavity, the flap was closed with 5/0 sutures (Supramid®, B. Braun, Rubí, Barcelona, Spain). In all cases 0.12% chlorhexidine rinses twice a day for 10 days were prescribed, along with 600 mg ibuprofen three times a day as needed. The sutures were removed 7 days after surgery.

- Evaluation of the tomographic parameters in CBCT

The buccal cortical bone measurements were made preoperatively (T0) and one year after apical surgery (T1). All measurements were made by the same calibrated examiner (AB). An analysis was made of the influence of patient age and gender, tooth location (maxillary or mandibular) and type of tooth (incisors, canines, premolars or molars) upon the changes in the measurements.

The tomographic images were obtained using a Planmeca scanner (ProMax 3D classic, Helsinki, Finland) with the Planmeca Romexis Viewer 4.5.2 application, and the analyses were made directly on the computer screen (resolution 1280 x 1024 pixels). Sagittal cross-sections were used for measuring the buccal cortical bone, and in the case of teeth with multiple roots (mesial and distal roots) one section was made for each affected root, for individual (independent) study.

**Figure 1:**
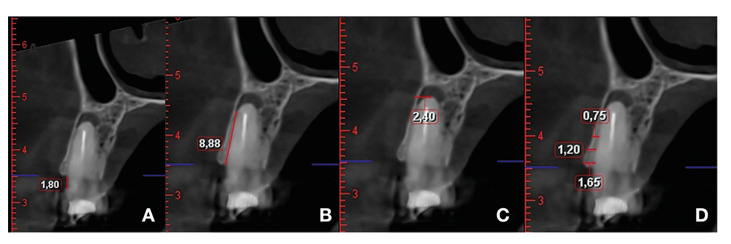
Measurements of the vestibular cortex before surgery and after one year after follow-up. (A: distance CEJ-BBC (difference between the two time points were the bone loss); B: vestibular cortical height or BCL (from the bone crest to the start of the lesion); C: depth of the apex with respect to the vestibular cortex or AD (from the medial position of the root to the vestibular bone cortex) and D: width of the vestibular cortex or BCT at 1-3-5 mm from the vestibular bone crest or COV (from the root perpendicular to the cortex)).

Intra-examiner reproducibility was assessed by measuring CEJ-BBC in 17 randomly selected teeth separated by an interval of four weeks. The mean variability between the repeated measurements showed a good concordance (intraclass correlation coefficient [ICC] = 0.0994) and variability coefficient (VC = 3.6%). These results evidenced good reproducibility of the measurements. The following measurements were made (Fig. 1):

(A) Distance from the cementoenamel junction (CEJ) to the buccal bone crest (CEJ-BBC); the difference in this parameter between the two timepoints (T0 and T1) yielded the marginal bone loss of the tooth.

(B) Height from the buccal cortical bone crest to the start of the lesion (BCL). The possible presence of buccal cortical fenestration was assessed, and its size was measured.

(C) Apical depth to the buccal cortical bone (AD): this was defined as the distance from the apex at its middle portion to the buccal cortical bone.

(D) Buccal cortical bone thickness (BCT): this was defined as the distance measured perpendicular to the axis of the tooth at 1.3 and 5 mm from the buccal bone crest.

The tomographic measurements were related to each other.

The calculation of volume from the CBCT scans was made using the Planmeca Romexis Viewer 4.5.2 application, and the size of the area was calculated in manual mode. The program automatically converts the measurements from voxels to mm3. In the follow-up images obtained after one year, those in which the periodontal width did not exceed twice the space were recorded as 0 mm3 defects (healed case). For obtaining the measurements in the posterior areas (molars and premolars), use was made of the coronal sections of the CBCT scans, while in the anterior areas (incisors and canines), use was made of the axial sections.

The healing evaluation was based on the classification of the “Modified Penn 3D criteria” scale. The results were classified as success (for cases assigned to the complete or limited healing categories) or failure (for cases assigned to the uncertain or unsatisfactory healing categories) (23).

- Statistical analysis

The study parameters and differences were reported as the means, standard deviation (SD) and median. In view of the absence of normal data distribution, nonparametric tests were used. The Wilcoxon test was performed to assess changes in the periodontal and radiographic parameters from pre- to postsurgery. The Mann-Whitney U-test in turn was used to analyze differences in distributions according to gender or type of tooth. The significance level was set at 5% (*p*=0.05). All analyses were performed using the SPSS version 15.0 statistical package (IBM Corp., Somers, NY, USA).

## Results

Between January 2020 and December 2022, a total of 125 patients underwent periapical surgery for the treatment of chronic periapical lesions. Thirty-three patients were excluded from the study: 4 minor patients, 3 cases of teeth with endo-periodontal lesions, 4 cases of apical cracks, 4 cases because the bone cavity was filled with a bone substitute, and 1 case because a resorbable membrane was used, 2 cases where the palatal root was affected and/or the surgical access was better through the palate, 9 patients had a lack of data collection and 6 cases presented blurred images and/or artifacts that prevented visualization of the tooth under study. A total of 92 patients were thus finally included in the study: 35 males (38%) and 57 females 62%), with a mean age of 46.5 ± 15.6 years (range 18-82) and involving 92 periapical surgeries.

Each patient contributed one tooth to the investigation, representing a total of 92 teeth (68 maxillary and 24 mandibular teeth) with a total of 139 roots of which 111 of them presented lesions.

- Tomographic Measurements:

The study evaluated the dimensions of buccal cortical bone and lesion volume using CBCT scans, with results summarized in Table 1. After periapical surgery, the distance from the cementoenamel junction to the buccal bone crest (CEJ-BBC) increased from 2.39 mm to 2.62 mm, reflecting a marginal bone loss of 0.23 ± 0.87 mm (*p*=0.001). Although both arches exhibited a similar average variation (+0.23 mm), measurements were significantly greater in the maxilla (*p*=0.959).

The height from the buccal cortical bone crest to the start of the lesion (BCL) decreased by 24.5% (1.50 ± 2.39 mm) post-surgery (*p*<0.001). Initially, 25.2% of cases showed fenestrations with a mean size of 2.20 ± 0.91 mm, but this reduced to 14.4% with a mean size of 3.23 ± 1.75 mm one year later (*p*<0.001). Fenestrations were more common in maxillary teeth (33.3% at T0 versus 15.4% at T1) than in mandibular teeth (6.1% at T0 versus 12.1% at T1) (*p*=0.004).

The apical depth measured 4.29 ± 2.05 mm and decreased by an average of 0.73 ± 1.02 mm (27%) one year after surgery. Buccal cortical bone thickness (BCT) decreased by an average of 0.23, 0.30, and 0.36 mm at 1, 3, and 5 mm from the bone crest, respectively (*p*<0.05). Significant differences were observed in anterior versus posterior teeth, especially at the 1 mm measurement, with greater reductions noted in posterior teeth. Initial BCT was a predictor of volume reduction, and at T1, greater thickness correlated with less volume variation.

Statistical correlations revealed that at T0, CEJ-BBC was inversely related to BCT at 3 and 5 mm (r= -0.21 and r= -0.22). BCL positively correlated with apical depth (AD) (r= 0.83; *p*<0.001). At T1, less fenestration was noted at 3 mm thickness (OR=0.42; *p*=0.048), with similar inverse relationships between CEJ-BBC and BCT at 3 and 5 mm (r= -0.25 and r= -0.27) and between CEJ-BBC and AD (r= -0.22). The variation in CEJ-BBC was inversely related to changes in BCT (*p*=0.031).

The mean lesion volume decreased from 305 ± 589 mm³ (median 83 mm³) at T0 to 27 ± 92 mm³ at one year post-surgery, representing a significant reduction of 91.1%. Greater volume reduction occurred in anterior teeth compared to posterior ones (*p*=0.052). Although age did not statistically affect volume reduction, a trend indicated younger patients experienced greater decreases (*p*=0.073).

The overall success rate after one year was 88%. Tooth position significantly influenced outcomes (*p*=0.007), with molars showing the lowest success rate (73.3%). Additionally, the number of roots involved correlated with healing rates, where single-rooted teeth had a 95% success rate, and those with one affected root achieved a 91.5% healing rate.

No tomographic variables at T0 predicted final healing; however, certain post-operative factors emerged as predictors: the presence of fenestration reduced healing probability by 74% (OR=0.26; *p*=0.025), and larger lesion volumes similarly decreased healing chances (OR=0.98; *p*<0.001), with each additional 1 mm³ of lesion volume decreasing healing probability by 2%.

## Discussion

The present study analyzed buccal cortical bone and lesion volume using CBCT in teeth with persistent periapical disease, comparing measurements before and one year after periapical surgery. Significant dimensional changes in the buccal cortical bone were observed post-surgery, although these were not clinically relevant. Larger lesion volumes and the presence of fenestrations were shown to decrease healing rates, with an overall success rate of 88%.

Evaluating buccal alveolar bone can be challenging due to its small thickness, necessitating precise measurement systems like CBCT, the most accepted technique for this analysis (4). Most CBCT studies focus on diagnostic measurements for healthy teeth (7-11). Notably, previous research has not monitored three-dimensional changes in buccal cortical bone after apical surgery, with the only relevant longitudinal study by von Arx *et al*. (12). Our study compared preoperative and postoperative measurements with those from healthy teeth (7-11) and clinically recorded measurements on the day of surgery (24) to assess differences.

The distance from the amelocemental junction to the buccal bone crest (CEJ-BBC) varies based on tooth location. Nahaas and Naiem (8) reported averages of 2.1 mm for central incisors and 2.09 mm for lateral incisors, while Rojo-Sanchis *et al*. (9) found averages of 2.34 mm for first premolars and 1.82 mm for second premolars. In teeth with pathology, both von Arx *et al*. (12) and this study found slightly higher averages (2.92 mm and 2.51 mm, respectively), but these differences were not clinically significant. Our findings showed greater marginal bone loss compared to von Arx *et al*. (12), but again without clinical relevance (0.23 mm versus 0.15 mm).

Regarding the height from the buccal cortical bone crest to the lesion (BCL), Ramanauskaite *et al*. (13) noted that diseased teeth had shorter distances compared to healthy ones. In our study, the mean BCL was 7.26 mm, exceeding the 4.73 mm reported by von Arx *et al*. (24) measured clinically. Kreisler *et al*. (25) and Song *et al*. (26) identified buccal bone plate height as a significant predictor of healing, while Kim *et al*. (19) disagreed, suggesting that this height is not a predictor.

Fenestrations smaller than 3 mm may not be easily detecTable via CBCT, as noted by Kopacz *et al*. (27), who found only three of seven clinically detected fenestrations visible in CBCT. In our study, 28 coinciding perforations were noted at baseline, with an average size of 2.20 mm, which reduced the probability of healing by 74% at follow-up (OR=0.26; *p*=0.025).

The apical depth relative to the buccal cortical bone was clinically measured by von Arx *et al*. (24), yielding a mean value of 4.44 mm. In this study, CBCT measurements yielded a similar value of 4.29 mm, which did not correlate with healing outcomes.

Buccal cortical bone thickness (BCT) has two reference points: the cementoenamel junction (CEJ) (10,12,14,24) or the buccal bone crest (BBC) (7-10). We chose to use BBC for reliability, given that CEJ can be affected by prosthetic restorations. One year post-surgery, marginal bone loss was evident, impacting BBC and resulting in BCT measurements of 1.13, 2.06, and 3.04 mm at baseline, decreasing to 0.90, 1.76, and 2.68 mm respectively after one year. Notably, Zekry *et al*. (16) suggested that bone thickness tends to be greater in females, while Wang *et al*. (15) found that older age correlates with reduced buccal cortical bone thickness. In our study, no significant associations were found between patient age and gender; however, a trend indicated greater volume reduction in younger individuals. Additionally, a significant relationship was noted between BCT and volume reduction, with greater width linked to lesser volume reduction.

Rojo-Sanchis *et al*. (9) noted an inverse relationship between CEJ-BBC distance and BCT in anterior maxillary teeth, which was consistent with our findings at both T0 and T1. A positive relationship was also observed between BCL and apical depth at both time points.

The preoperative lesion volume is crucial for periapical surgery outcomes. Kim *et al*. (19) identified a lesion volume over 50 mm³ as a significant negative predictor of outcomes (*p*=0.028). Our initial median lesion volume was 83 mm³. While initial volume did not influence healing, the tooth's position significantly impacted the reduction of lesion volume, with less reduction noted in posterior teeth, aligning with findings by Ramis-Alario *et al*. (20). Post-surgery, Curtis *et al*. (28) reported a 95% reduction in lesion volume over 48 months with a success rate of 92.2%, while Schloss *et al*. (23) noted an 83.7% success rate after 37 months. Ramis-Alario *et al*. (20) documented a mean preoperative volume of 147.7 mm³ decreasing to 15.5 mm³, corresponding to a healing rate of 6.2 mm³ per month, with a 93% success rate after two years. Kreisler *et al*. (25) found an 88% success rate after 12 months, with females achieving slightly higher rates than males. Our one-year follow-up showed a 91.1% decrease in lesion volume, more pronounced in teeth with thinner buccal cortical bone. Location was the only variable statistically significant in influencing healing outcomes, with our study's success rate aligning with Kim *et al*. (19) but lower than others (20,26), as success was based solely on tomographic criteria.

Limitations of this study include a small sample size, highlighting the need for further research with larger cohorts and extended follow-up periods.

## Conclusions

The buccal cortical bone did not undergo any relevant clinical changes one year after periapical surgery. The lesion volume was reduced by 91.1% at 12 months after surgery, with a greater reduction in anterior teeth than in posterior teeth. A greater width of the buccal cortical bone, a smaller reduction in lesion volume. The success rate at one year after apical surgery was 88%, but posterior teeth and teeth with more involved roots in the lesion had a worse success rate. One year after surgery, a larger lesion volume as well as the existence of fenestrations in the buccal cortical bone decreased the healing.

## Figures and Tables

**Table 1:** Tomographic measurements of the vestibular table dimensions at T0, T1 and T1-T0 changes for total sample and for tooth position. Mean ± SD (median) in mm for linear dimensions and in mm3 for volume of the lesion.

Measurements		N	Pre-surgery (T0)	1 year (T1)	*p value*
Distance CEJ-BBC	Total	111	2.39 ± 1.07	2.62 ± 1.06	0.001**
	MX	78	2.58 ± 1.08	2.81 ±1.05	<0.05*
	MD	33	1.94 ± 0.91	2.17 ± 0.97	<0.05*
Buccal cortical bone height	Total	111	7.26 ± 2.24	5.75 ± 2.21	<0.001***
	MX	78	7.31 ± 2.35	5.78 ± 2.18	<0.05*
	MD	33	7.12 ± 2.01	5.70 ± 2.33	<0.05*
Apex depth	Total	111	4.29 ± 2.05	3.56 ± 1.98	<0.001***
	MX	78	3.19 ± 2.22	2.73 ± 3.10	<0.05*
	MD	33	3.89 ± 2	2.99 ± 1.68	<0.05*
Buccal cortical thickness at 1mm	Total	111	1.13 ± 0.71	0.90 ± 0.42	<0.001***
	MX	78	1.18 ± 0.76	0.92 ± 0.45	<0.05*
	MD	33	1.01 ± 0.54	0.85 ± 0.34	<0.05*
Buccal cortical thickness at 3mm	Total	111	2.06 ± 0.78	1.76 ± 0.61	<0.001***
	MX	78	2.10 ± 0.83	1.75 ±0.64	<0.05*
	MD	33	1.95 ± 0.65	1.77 ± 0.53	<0.05*
Buccal cortical thickness at 5mm	Total	111	3.04 ± 0.81	2.68 ± 0.73	<0.001***
	MX	78	3.08 ± 0.87	2.64 ± 0.81	<0.05*
	MD	33	2.95 ± 0.65	2.77 ± 0.51	<0.05*
Volume of the lesion	Total	92	305 ± 589	27 ± 92	<0.001***
	MX	68	328 ± 636	24 ± 62	<0.001***
	MD	24	434 ± 2	39 ± 151	<0.001***
Success rate (%)	Total	92	-	88	-
	MX	68	-	89.7	-
	MD	24	-	83.3	-

N: number of teeth. CEJ-BBC: cemelocemental line-vestibular bone crest. BCL: height of the vestibular cortex up to the lesion. AD: depth of the apex to the vestibular cortex. BCT: width of the vestibular cortex. MX: maxilla. MD: mandible. I: incisor. C: canine. PM: premolar. M: molar.
